# KRAS Affects Adipogenic Differentiation by Regulating Autophagy and MAPK Activation in 3T3-L1 and C2C12 Cells

**DOI:** 10.3390/ijms222413630

**Published:** 2021-12-20

**Authors:** Wenjie Yu, Cheng-Zhen Chen, Yanxia Peng, Ze Li, Yan Gao, Shuang Liang, Bao Yuan, Nam-Hyung Kim, Hao Jiang, Jia-Bao Zhang

**Affiliations:** 1 Department of Laboratory Animals, Jilin Provincial Key Laboratory of Animal Model, Jilin University, Changchun 130062, China; yuwj19@mails.jlu.edu.cn (W.Y.); chencz@jlu.edu.cn (C.-Z.C.); pengyx18@mails.jlu.edu.cn (Y.P.); lize20@mails.jlu.edu.cn (Z.L.); gyan08@163.com (Y.G.); liangshuang85@jlu.edu.cn (S.L.); yuan_bao@jlu.edu.cn (B.Y.); nhkim@chungbuk.ac.kr (N.-H.K.); 2 Department of Animal Science, Chungbuk National University, Cheongju 361-763, Chungbuk, Korea

**Keywords:** KRAS, adipogenic differentiation, lipid accumulation, autophagy, MAPK, PI3K

## Abstract

Kirsten rat sarcoma 2 viral oncogene homolog (*Kras*) is a proto-oncogene that encodes the small GTPase transductor protein KRAS, which has previously been found to promote cytokine secretion, cell survival, and chemotaxis. However, its effects on preadipocyte differentiation and lipid accumulation are unclear. In this study, the effects of KRAS inhibition on proliferation, autophagy, and adipogenic differentiation as well as its potential mechanisms were analyzed in the 3T3-L1 and C2C12 cell lines. The results showed that KRAS was localized mainly in the nuclei of 3T3-L1 and C2C12 cells. Inhibition of KRAS altered mammalian target of rapamycin (*Mtor*), proliferating cell nuclear antigen (*Pcna*), *Myc*, peroxisome proliferator-activated receptor γ (PPARγ), CCAAT/enhancer binding protein beta (*C/ebp-β*), diacylglycerol O-acyltransferase 1 (*Dgat1*), and stearoyl-coenzyme A desaturase 1 (*Scd1*) expression, thereby reducing cell proliferation capacity while inducing autophagy, enhancing differentiation of 3T3-L1 and C2C12 cells into mature adipocytes, and increasing adipogenesis and the capacity to store lipids. Moreover, during differentiation, KRAS inhibition reduced the levels of extracellular regulated protein kinases (ERK), c-Jun N-terminal kinase (JNK), p38, and phosphatidylinositol 3 kinase (PI3K) activation. These results show that KRAS has unique regulatory effects on cell proliferation, autophagy, adipogenic differentiation, and lipid accumulation.

## 1. Introduction

Obesity is a chronic metabolic disease caused by excessive fat accumulation [1,2]. It significantly increases the risk of type 2 diabetes, fatty liver, atherosclerosis, and other diseases, resulting in reduced quality of life and life span [3,4,5]. Adipose tissue metabolism exerts a profound impact on whole-body metabolism. Energy imbalance leads to storage of excess energy in adipocytes, which contributes to the increased adipose tissue mass in obesity [6]. One-third of adipose tissue is composed of mature adipocytes, and the remainder is composed of a heterogeneous collection of cells, such as fibroblasts, mesenchymal stem cells (MSCs), and endothelial progenitor cells (EPCs) [7]. Increases in adipocyte numbers are caused by certain cytokines and signaling molecules that induce MSCs to be converted into preadipocytes, which later differentiate into adipocytes [8]. Adipogenic differentiation of preadipocytes with concomitant increases in adipocyte size and number eventually leads to excessive lipid accumulation and obesity [9].

The 3T3-L1 preadipocyte cell line and the C2C12 myoblast cell line are widely used in adipocyte-related studies focusing on adipogenic differentiation and adipogenic transdifferentiation. They have also been used to explore the effects of functional genes [10,11,12] and compounds such as permethrin [13], stearidonic acid [14], and curcumin [15] on adipogenesis as well as to explore the mechanisms of triglyceride (TG) formation and accumulation and the treatment of obesity-related diseases. In an appropriate microenvironment and under suitable gene expression conditions, preadipocytes undergo clonal expansion and subsequent terminal differentiation. During adipogenesis, fibroblast-like preadipocytes differentiate into lipid-laden and insulin-responsive adipocytes [16]. It is known that peroxisome proliferator-activated receptor gamma (PPARγ) and CCAAT/enhancer-binding proteins (C/EBPs) are the major determinants of adipocyte fate [17,18]. C/EBP-β is induced early to transactivate the expression of C/EBP-α and PPARγ, two master transcription factors for terminal adipocyte differentiation [19,20], and loss of C/EBP-β activity manifests as a loss of differentiation-induced C/EBP-α and PPARγ expression and a dramatic reduction in lipid accumulation. Mitogen-activated protein kinases (MAPKs) and autophagy pathways, which play very important roles in cell proliferation, autophagy, and differentiation, also contribute to adipogenic differentiation [21,22,23,24,25].

Kirsten rat sarcoma 2 viral oncogene homolog (*Kras*) is a proto-oncogene that encodes a small GTPase transductor protein called KRAS, which has previously been found to promote cytokine secretion, cell survival, and chemotaxis [26,27]. It is also a signal transducer protein that plays important regulatory roles in various cellular signaling pathways. Previous studies have indicated that KRAS mediates the reprogramming of glutamine metabolism [28] and controls the Hedgehog [29], phosphatidylinositol 3 kinase (PI3K) [30], CTEN [31], RAL-GEF [26], and Nrf2 [32] pathways, thereby acting as a key regulatory factor and a therapeutic target for cancers [33,34]. In addition, stimulation by appropriate ligands induces a signaling cascade, linking membrane-associated tyrosine kinase receptors to MAPK proteins, that involves the Ras protein [35], which participates in the regulation of lipogenesis. KRAS belongs to the Ras superfamily, which is a group of guanosine triphosphate (GTP)-binding proteins [26]; thus, KRAS may affect preadipocyte proliferation and differentiation by regulating MAPK. However, few studies have clarified the key roles of KRAS in preadipocyte differentiation and lipid accumulation.

In this study, we explored the roles of KRAS in autophagy, proliferation, adipogenic differentiation, and lipid accumulation in the 3T3-L1 and C2C12 cell lines. We also analyzed the phosphorylation levels of extracellular regulated protein kinases (ERK), c-Jun N-terminal kinase (JNK), p38, and PI3K. The results of this study will help elucidate the mechanisms of preadipocyte differentiation, adipocyte maturation, lipid accumulation, and obesity.

## 2. Results

### 2.1. KRAS Is Expressed Mainly in 3T3-L1 and C2C12 Cell Nuclei

**Figure 1:**
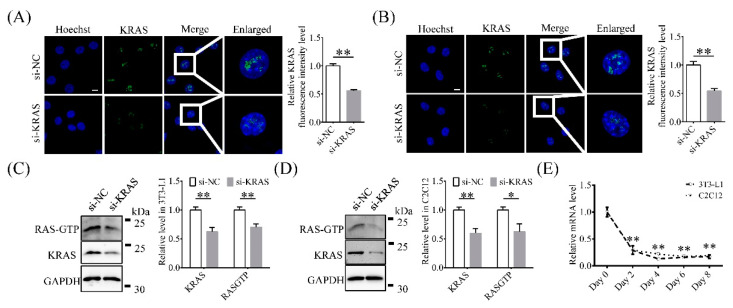
Intracellular localization of Kirsten rat sarcoma 2 viral oncogene homolog (KRAS) and knockdown efficiency determination. (**A**,**B**) Representative immunofluorescence images and relative fluorescence intensity levels of KRAS in 3T3-L1 and C2C12 cells with or without KRAS inhibition. Scale bar = 10 μm. Significant differences are represented with ** (*p* < 0.01). (**C**,**D**) Protein levels of KRAS and RAS-guanosine triphosphate (GTP) in 3T3-L1 and C2C12 cells with or without siRNA treatment. Significant differences are represented with * (*p* < 0.05) and ** (*p* < 0.01). (**E**) Relative *Kras* mRNA expression level changes during adipogenic differentiation in 3T3-L1 and C2C12 cells. Significant differences are represented with ** (*p* < 0.01).

The immunofluorescence results showed that KRAS was localized in the nuclei of 3T3-L1 and C2C12 cells (Figure 1A,B). Compared to those in the negative control (NC) group, the fluorescence intensity levels of KRAS in 3T3-L1 and C2C12 cells with KRAS inhibition were significantly lower (0.56 ± 0.02 times and 0.54 ± 0.04 times the values in the NC group, respectively; Figure 1A,B). In addition, the protein levels of KRAS and RAS-GTP in 3T3-L1 cells with KRAS inhibition were 0.62 ± 0.08-fold and 0.70 ± 0.06-fold of those in the NC group, respectively (Figure 1C), while the protein levels in C2C12 cells with KRAS inhibition were 0.60 ± 0.09-fold and 0.63 ± 0.13-fold of those in the NC cells, respectively (Figure 1D). The level of KRAS mRNA gradually decreases with the adipogenic differentiation (from day 2) of the cells (Figure 1E). These results indicated that KRAS was localized in the nuclei and its inhibition reduced the level of KRAS and RAS-GTP in 3T3-L1 and C2C12 cells under normal physiological conditions.

### 2.2. Inhibition of KRAS Enhanced Adipogenic Differentiation and Adipogenesis

**Figure 2:**
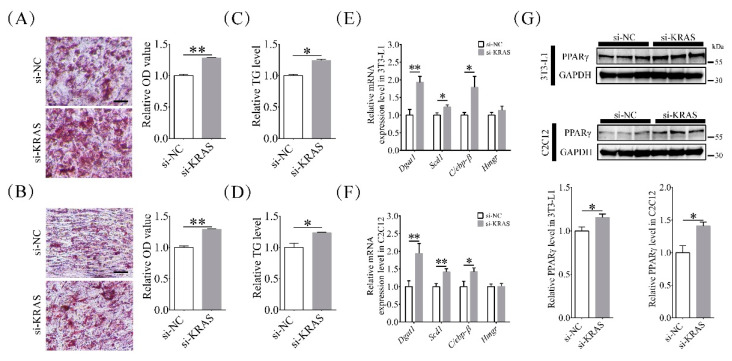
Effects of KRAS on adipogenic differentiation and lipid accumulation in 3T3-L1 and C2C12 cells. (**A**,**B**) Representative oil red O (ORO) staining images and relative ORO optical density (OD) value (OD_520nm_ values to total protein level) changes in 3T3-L1 and C2C12 cells after induction of adipogenic differentiation with or without KRAS inhibition. The ORO staining images suggested that the inhibition of KRAS enhanced the lipid accumulation after induction of adipogenic differentiation in 3T3-L1 and C2C12 cells. Scale bar = 50 μm. Significant differences are represented with ** (*p* < 0.01). (**C**,**D**) Relative triglyceride (TG) level changes in 3T3-L1 and C2C12 cells after induction of adipogenic differentiation with or without KRAS inhibition. Significant differences are represented with * (*p* < 0.05). (**E**,**F**) mRNA expression of diacylglycerol O-acyltransferase 1 (*Dgat1)*, stearoyl-coenzyme A desaturase 1 (*Scd1)*, CCAAT/enhancer binding protein beta *(C/ebp-β)*, and 3-hydroxy-3-methylglutaryl-coenzyme A reductase *(Hmgr)* in 3T3-L1 and C2C12 cells before induction of adipogenic differentiation. Significant differences are represented with * (*p* < 0.05) and ** (*p* < 0.01). (**G**) Relative peroxisome proliferator-activated receptor γ (PPARγ) protein level changes in 3T3-L1 and C2C12 cells after induction of adipogenic differentiation. Significant differences are represented with * (*p* < 0.05).

In order to explore the effects of KRAS on adipogenic differentiation and lipid accumulation, we detected the level of triglyceride (TG), relative optical density (OD) value of oil red O (ORO), and the expression level of adipogenesis-related molecules PPARγ, *Dgat1*, *Scd1*, *C/ebp-β*, and 3-hydroxy-3-methylglutaryl-coenzyme A reductase (*Hmgr*) after KRAS inhibition. As shown in Figure 2A,C, compared to the NC group, the si-KRAS-treated group of 3T3-L1 cells had significantly higher ORO OD values and TG levels (1.28 ± 0.01 times and 1.24 ± 0.02 times those in the NC group, respectively). Compared to those in the NC cells, the ORO OD values and TG levels in C2C12 cells with KRAS inhibition were significantly higher (1.29 ± 0.01 times and 1.24 ± 0.01 times the values in the NC group, respectively; Figure 2B,D). Inhibition of KRAS enhanced the lipid accumulation after induction of adipogenic differentiation in both 3T3-L1 and C2C12 cells. In addition, the mRNA levels of *Dgat1*, *Scd1*, *C/ebp-β*, and *Hmgr* in 3T3-L1 cells with KRAS inhibition were 1.94 ± 0.17-fold, 1.23 ± 0.06-fold, 1.79 ± 0.31-fold, and 1.14 ± 0.12-fold of those in the NC group, respectively (Figure 2E), while the mRNA levels of these genes in C2C12 cells with KRAS inhibition were 1.94 ± 0.28-fold, 1.42 ± 0.09-fold, 1.42 ± 0.10-fold, and 1.01 ± 0.08-fold of those in the NC cells, respectively (Figure 2F). The protein levels of PPARγ in 3T3-L1 cells with KRAS inhibition were 1.16 ± 0.04-fold of those in the NC cells (Figure 2G), while the levels in C2C12 cells with KRAS inhibition were 1.41 ± 0.06-fold of those in the NC cells, respectively (Figure 2G). These results indicated that inhibition of KRAS enhanced adipogenic differentiation and adipogenesis in 3T3-L1 and C2C12 cells.

### 2.3. Inhibition of KRAS Reduced Preadipocyte Proliferation

**Figure 3:**
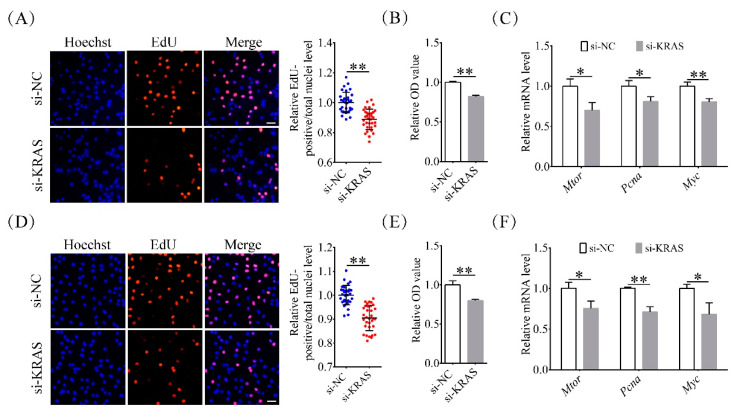
Effects of KRAS on cell proliferation in 3T3-L1 and C2C12 cells. (**A**) Representative 5-ethynyl-2′-deoxyuridine (EdU) staining images and relative percentages of EdU-positive cells among 3T3-L1 cells with or without KRAS inhibition. Scale bar = 50 μm. Significant differences are represented with ** (*p* < 0.01). (**B**) Cell proliferation capacity assay results for 3T3-L1 cells with or without KRAS inhibition. Significant differences are represented with ** (*p* < 0.01). (**C**) mRNA expression of mammalian target of rapamycin *(Mtor)*, proliferating cell nuclear antigen *(Pcna)*, and *Myc* in 3T3-L1 cells with or without KRAS inhibition. Significant differences are represented with * (*p* < 0.05) and ** (*p* < 0.01). (**D**) Representative EdU staining images and relative percentages of EdU-positive cells among C2C12 cells with or without KRAS inhibition. Scale bar = 50 μm. Significant differences are represented with ** (*p* < 0.01). (**E**) Cell proliferation capacity assay results for C2C12 cells with or without KRAS inhibition. Significant differences are represented with ** (*p* < 0.01). (**F**) mRNA expression of *Mtor*, *Pcna,* and *Myc* in C2C12 cells with or without KRAS inhibition. Significant differences are represented with * (*p* < 0.05) and ** (*p* < 0.01).

To explore the effects of KRAS on cell proliferation, the proportion of 5-ethynyl-2′-deoxyuridine (EdU)-positive cells was assayed among 3T3-L1 and C2C12 cells after KRAS inhibition. EdU, a DNA replication precursor analogue, has been widely used to monitor DNA synthesis and cell proliferation potentials [36]. Among 3T3-L1 cells, the proportion of EdU-positive nuclei in the KRAS inhibition group was 0.89 ± 0.07-fold of that in the NC group (Figure 3A). The CCK-8 (Cell Counting Kit-8) assay results also showed that the proliferation capacity in the KRAS inhibition group was 0.82 ± 0.02-fold of that in the NC group (Figure 3B). The mRNA levels of mammalian target of rapamycin *(Mtor)*, proliferating cell nuclear antigen *(Pcna)*, and *Myc* in the KRAS inhibition group were 0.70 ± 0.09-fold, 0.81 ± 0.06-fold, and 0.81 ± 0.04-fold of those in the NC group, respectively (Figure 3C).

Among C2C12 cells, the proportion of EdU-positive nuclei in the KRAS inhibition group was 0.90 ± 0.05-fold of that in the NC group (Figure 3D). The CCK-8 assay results also showed that the proliferation capacity in the KRAS inhibition group was 0.80 ± 0.02-fold of that in the NC group (Figure 3E). The mRNA levels of *Mtor*, *Pcna*, and *Myc* in the KRAS inhibition group were 0.75 ± 0.09-fold, 0.71 ± 0.06-fold, and 0.68 ± 0.15-fold of those in the NC group, respectively (Figure 3F). These results indicated that inhibition of KRAS reduced cell proliferation in 3T3-L1 and C2C12 cells.

### 2.4. Inhibition of KRAS Enhanced Preadipocyte Autophagy

**Figure 4:**
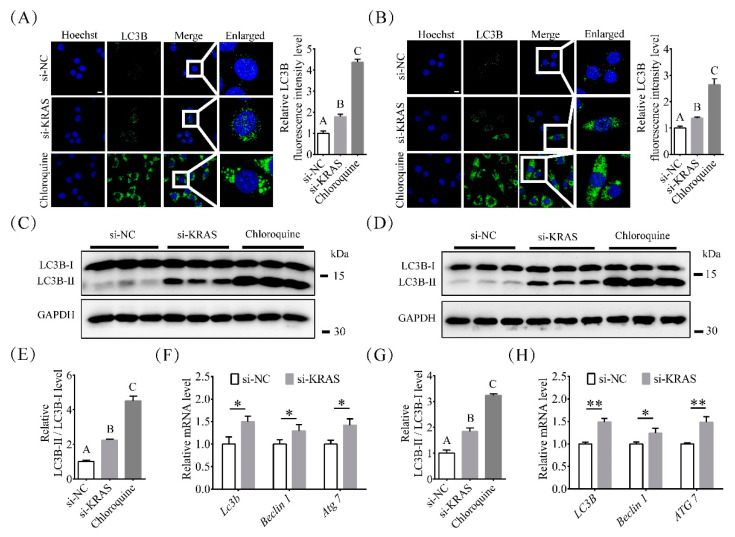
Effects of KRAS on autophagy in 3T3-L1 and C2C12 cells. (**A**,**B**) Representative immunofluorescence images and relative fluorescence intensity levels of microtubule-associated protein 1 light chain-3B (LC3B) in 3T3-L1 and C2C12 cells. Scale bar = 10 μm. Significant differences are represented with different capital letters (*p* < 0.01). (**C**,**E**) LC3B protein levels detected by Western blot analysis in 3T3-L1 cells. Significant differences are represented with different capital letters (*p* < 0.01). (**F**) mRNA expression of *Lc3b*, *Beclin1*, and autophagy related 7 (*Atg7)* in 3T3-L1 cells with or without KRAS inhibition. Significant differences are represented with * (*p* < 0.05). (**D**,**G**) LC3B protein levels detected by Western blot analysis in C2C12 cells. Significant differences are represented with different capital letters (*p* < 0.01). (**H**) mRNA expression of *Lc3b*, *Beclin1*, and *Atg7* in C2C12 cells with or without KRAS inhibition. Significant differences are represented with * (*p* < 0.05) and ** (*p* < 0.01).

Autophagy, one of the most important processes in cells, also plays important roles in adipogenesis [37]. Therefore, we detected the levels of microtubule-associated protein 1 light chain-3B (LC3B) in 3T3-L1 and C2C12 cells using immunofluorescence and Western blotting after transfection with siRNA. As shown in Figure 4A, among 3T3-L1 cells, the fluorescence intensity levels of LC3B in the si-KRAS and chloroquine-treated groups were 1.78 ± 0.11-fold and 4.37 ± 0.14-fold of those in the NC group, respectively. The protein levels of LC3B-II/LC3B-I in the si-KRAS and chloroquine-treated groups were 2.25 ± 0.06-fold and 4.51 ± 0.28-fold of those in the NC group (Figure 4C,E), respectively. The mRNA levels of *Lc3b*, *Beclin 1*, and, autophagy related 7 (*Atg7)* in 3T3-L1 cells with KRAS inhibition were 1.49 ± 0.13-fold, 1.30 ± 0.14-fold, and 1.42 ± 0.15-fold of those in the NC group, respectively (Figure 4F).

For C2C12 cells, the fluorescence intensity levels of LC3B in the si-KRAS and chloroquine-treated groups were 1.38 ± 0.04-fold and 2.64 ± 0.23-fold of those in the NC group (Figure 4B), respectively. The protein levels of LC3B-II/LC3B-I in the si-KRAS and chloroquine-treated groups were 1.84 ± 0.14-fold and 3.24 ± 0.06-fold of those in the NC group (Figure 4D,G), respectively. The mRNA levels of *Lc3b*, *Beclin 1*, and *Atg7* in C2C12 cells with KRAS inhibition were 1.49 ± 0.08-fold, 1.30 ± 0.14-fold, and 1.42 ± 0.15-fold of those in NC cells, respectively (Figure 4H). These results indicated that inhibition of KRAS enhanced autophagy in 3T3-L1 and C2C12 cells.

### 2.5. Inhibition of KRAS Decreased PI3K Phosphorylation Levels

**Figure 5:**
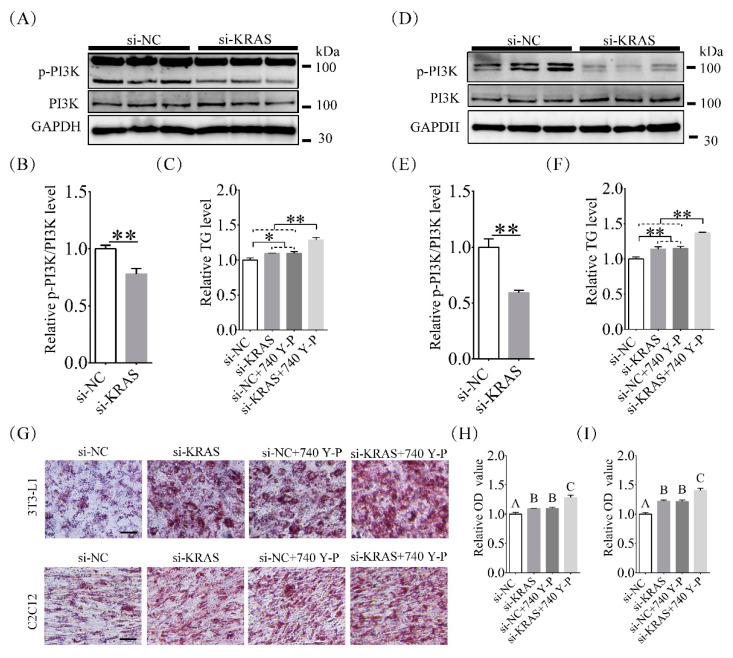
Effects of KRAS on phosphatidylinositol 3 kinase (PI3K) phosphorylation levels in 3T3-L1 and C2C12 cells. (**A**,**B**) Total and phosphorylated PI3K levels detected by Western blot analysis in 3T3-L1 cells. Significant differences are represented with ** (*p* < 0.01). (**C**) Relative TG levels in 3T3-L1 cells treated with si-NC, si-KRAS, si- negative control (NC) + 740 Y-P, and si-KRAS + 740 Y-P. Significant differences are represented with * (*p* < 0.05) and ** (*p* < 0.01). (**G**,**H**) The representative ORO staining images and relative ORO OD values showed the different lipid accumulations in 3T3-L1 cells treated with si-NC, si-KRAS, si-NC + 740 Y-P, and si-KRAS + 740 Y-P after induction of adipogenic differentiation. Scale bar = 50 μm. Significant differences are represented with different capital letters (*p* < 0.01). (**D**,**E**) Total and phosphorylated PI3K levels detected by Western blot analysis in C2C12 cells. Significant differences are represented with ** (*p* < 0.01). (**F**) Relative TG levels in C2C12 cells treated with si-NC, si-KRAS, si-NC + 740 Y-P, and si-KRAS + 740 Y-P. Significant differences are represented with ** (*p* < 0.01). (**G**,**I**) The representative ORO staining images and relative ORO OD values showed the different lipid accumulations in C2C12 cells treated with si-NC, si-KRAS, si-NC + 740 Y-P, and si-KRAS + 740 Y-P after induction of adipogenic differentiation. Scale bar = 50 μm. Significant differences are represented with different capital letters (*p* < 0.01).

Because of the essential effects of PI3K on lipid accumulation [38,39,40], we detected total PI3K and phosphorylated PI3K levels after KRAS inhibition. The results showed that the total levels of PI3K did not differ significantly between the KRAS inhibition group and the NC group. However, phosphorylation of PI3K was significantly decreased after KRAS inhibition. The relative levels of phosphorylated PI3K versus total PI3K in cells with KRAS inhibition were 0.78 ± 0.05-fold and 0.59 ± 0.02-fold of those in normal 3T3-L1 (Figure 5A,B) and C2C12 (Figure 5D,E) cells, respectively. Then, we assayed the TG levels after treatment with the PI3K activator 740 Y-P. The results showed that for 3T3-L1 cells, the TG levels in the si-KRAS, si-NC + 740 Y-P, and si-KRAS + 740 Y-P groups were 1.09 ± 0.01-fold, 1.10 ± 0.02-fold, and 1.28 ± 0.04-fold of those in the NC group, respectively (Figure 5C,D). For C2C12 cells, the TG levels in the si-KRAS, si-NC+740 Y-P, and si-KRAS+740 Y-P groups were 1.14 ± 0.03-fold, 1.15 ± 0.03-fold, and 1.37 ± 0.01-fold of those in the NC group, respectively (Figure 5C,F). In addition, among 3T3-L1 cells, the ORO OD values in the si-KRAS, si-NC+ 740 Y-P, and si-KRAS + 740 Y-P groups were 1.09 ± 0.01-fold, 1.10 ± 0.02-fold, and 1.28 ± 0.04-fold of those in the NC group, respectively (Figure 5G,H). Among C2C12 cells, the ORO OD values in the si-KRAS, si-NC + 740 Y-P, and si-KRAS + 740 Y-P groups were 1.22 ± 0.02-fold, 1.22 ± 0.02-fold, and 1.40 ± 0.04-fold of those in the NC group, respectively (Figure 5G,I). These results indicated that inhibition of KRAS decreased PI3K phosphorylation levels in 3T3-L1 and C2C12 cells.

### 2.6. Inhibition of KRAS Decreased MAPK Phosphorylation Levels

**Figure 6:**
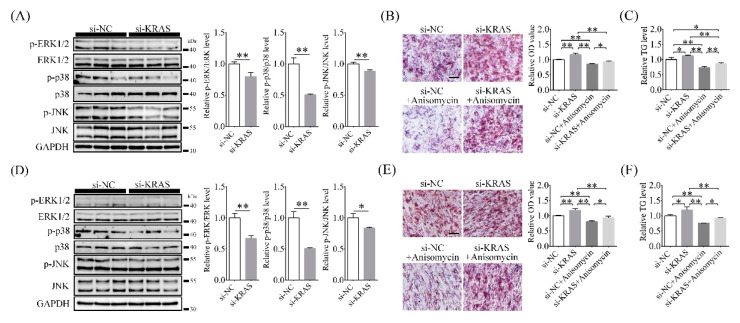
Effects of KRAS on mitogen-activated protein kinases (MAPK) activation in 3T3-L1 and C2C12 cells. (**A**) Changes in extracellular regulated protein kinases (ERK), c-Jun N-terminal kinase (JNK), and p38 phosphorylation levels in 3T3-L1 cells detected by Western blot analysis. Significant differences are represented with ** (*p* < 0.01). (**B**) The representative ORO staining images and relative ORO OD values showed the different lipid accumulations in 3T3-L1 cells with different treatments after induction of adipogenic differentiation. Scale bar = 50μm. Significant differences are represented with * (*p* < 0.05) and ** (*p* < 0.01). (**C**) The relative TG level changes in 3T3-L1 cells with different treatments. Significant differences are represented with * (*p* < 0.05) and ** (*p* < 0.01). (**D**) Changes in ERK, JNK, and p38 phosphorylation levels in C2C12 cells detected by Western blot analysis. Significant differences are represented with * (*p* < 0.05) and ** (*p* < 0.01). (**E**) The representative ORO staining images and relative ORO OD values showed the different lipid accumulations in C2C12 cells with different treatments after induction of adipogenic differentiation. Scale bar = 50 μm. Significant differences are represented with * (*p* < 0.05) and ** (*p* < 0.01). (**F**) The relative TG level changes in C2C12 cells with different treatments. Significant differences are represented with * (*p* < 0.05) and ** (*p* < 0.01).

MAPK signals also play important roles in adipogenesis and lipid accumulation [24,41]. Therefore, we investigated the activation of MAPK signals (as indicated by the phosphorylation levels of ERK, JNK, and p38) after KRAS inhibition. The results showed that compared with the NC group, the relative phosphorylated versus total protein levels of ERK, p38, and JNK in the KRAS inhibition group were 0.80 ± 0.07-fold, 0.51 ± 0.01-fold, and 0.89 ± 0.02-fold of those in the NC group for 3T3-L1 cells, respectively (Figure 6A). In C2C12 cells, they were 0.67 ± 0.05-fold, 0.87 ± 0.01-fold, and 0.84 ± 0.02-fold of those in the NC group, respectively (Figure 6C). Then, we assayed the ORO OD values after treatment with anisomycin (ANI, a MAPK activator). The results showed that for 3T3-L1 cells, the ORO OD values in the si-KRAS, si-NC + ANI, and si-KRAS + ANI groups were 1.17 ± 0.05-fold, 0.86 ± 0.02-fold, and 0.94 ± 0.02-fold of those in the NC group, respectively (Figure 6B). For C2C12 cells, the ORO OD values in the si-KRAS, si-NC + 740 Y-P, and si-KRAS + 740 Y-P groups were 1.19 ± 0.02-fold, 0.81 ± 0.02-fold, and 0.93 ± 0.06-fold of those in the NC group, respectively (Figure 6E).

Among 3T3-L1 cells, the TG levels in the si-KRAS, si-NC + ANI, and si-KRAS + ANI groups were 1.12 ± 0.02-fold, 0.74 ± 0.04-fold, and 0.86 ± 0.03-fold of those in the NC group, respectively (Figure 6C). Among C2C12 cells, the TG levels in the si-KRAS, si-NC+ANI and si-KRAS+ANI groups were 1.19 ± 0.10-fold, 0.76 ± 0.01-fold, and 0.92 ± 0.02-fold of those in the NC group, respectively (Figure 6F). These results indicated that inhibition of KRAS decreased MAPK phosphorylation levels in 3T3-L1 and C2C12 cells.

## 3. Discussion

TG stored in white adipose tissue is the major energy reserve in mammals and is also a hallmark substance with biological function in adipocytes. In this study, both 3T3-L1 and C2C12 cells were able to undergo adipogenic differentiation when KRAS was inhibited before inducing differentiation. Interestingly, TG levels were significantly increased after KRAS inhibition, indicating that KRAS plays important roles in enhancing the adipogenic differentiation and lipid accumulation in 3T3-L1 and C2C12 cells.

When adipogenesis is triggered, the nuclear architecture of preadipocytes undergoes substantial modifications. Among many other modifications, the nuclear lamina is reorganized, chromosome territories decondense and change their relative positions, and adipogenic genes, such as *C/ebp-β* and *Pparγ*, undergo repositioning. These modifications are accompanied by dramatic changes in chromatin accessibility, promoter occupancy, and super-enhancer formation [42,43,44]. Our study showed that KRAS was located in the nuclei of 3T3-L1 and C2C12 cells under normal physiological conditions, indicating that nuclear KRAS may mediate signaling that can activate/deactivate some transcription factors. This also suggests that KRAS may contribute to the expression of some adipogenic genes that might affect adipogenic differentiation during the division or differentiation of preadipocytes.

Our results also showed that the mRNA or protein levels of *C/ebp-β*, PPARγ, *Myc*, *Pcna*, and *Mtor* were significantly changed with KRAS inhibition. PPARγ binds to retinoid X receptors to regulate adipose differentiation and lipid metabolism after being coactivated by cytokines and molecular events [45]. As PPARγ is a potential inducer of adipocyte differentiation, this may explain why increasing PPARγ expression increases adipogenic differentiation and lipid accumulation in 3T3-L1 and C2C12 cells [46,47]. In addition, previous studies have indicated that in preadipocyte cell lines and primary preadipocytes, growth arrest is required for preadipocyte differentiation [48,49]. In this study, we found that specific KRAS inhibition before differentiation significantly increased the protein level of PPARγ and decreased the mRNA levels of *Myc*, *Pcna,* and *Mtor*, suggesting that KRAS inhibition leads to enhanced fibroblast growth arrest mediated by regulating PPARγ [50], *Myc* [51,52], PCNA [53,54], and mTOR [55,56]. These findings also suggest that KRAS inhibition promotes the differentiation of preadipocytes with growth arrest and low proliferation ability into mature adipocytes and regulates lipid homeostasis mediated by PPARγ [57]. In addition, the increase in *C/ebp-β* mediated by KRAS inhibition also indicates that KRAS affects differentiation and adipogenesis in 3T3-L1 and C2C12 cells [58,59,60]. Combined with previous findings showing that lipid accumulation is decreased in DGAT1- and SCD1-knockout mice [61,62] and an increase of SCD1 causes lipid accumulation [63,64], these results suggest that changes in KRAS expression lead to changes in lipid accumulation capacity.

Previous studies have shown that targeted deletion of autophagy-related genes impairs adipogenesis and reduces white adipose tissue (to only 20% of that in the wild type), while downregulating LC3B [25,65,66]. The presence of LC3B in autophagosomes and the conversion of LC3B-I to the lower migrating form of LC3B-II through lipidation by a ubiquity-like system involving Atg7 were used as indicators of autophagic processes, while Beclin-1 was also considered as essential in the induction process of autophagy [67,68,69,70,71]. Our results showed that inhibition of KRAS significantly increased the mRNA expression levels of *Atg7* and *Beclin 1*, the protein levels of LC3B, and the numbers of cytoplasmic autophagosomes in 3T3-L1 and C2C12 cells. These findings indicate that KRAS inhibition may affect the conversion of LC3B-I to LC3B-II through lipidation by a ubiquitin-like system involving Atg7 that allows LC3B to associate with autophagic vesicles [72,73]. Chloroquine inhibits the degradation of autophagosomes, thereby inhibiting autophagy levels in cells. During this process, the mRNA levels of *Lc3b*, *Atg7*, and *Beclin 1* will not increase. However, in this study, the inhibition of KRAS resulted in increased levels of *LC3b, Atg7,* and *Beclin 1*. Based on these results, we believe that inhibition of KRAS indeed increases the level of autophagy in cells, rather than preventing the degradation of autophagosomes and thus inhibiting autophagy in cells. Along with changes in PPARγ and *C/ebp-β* levels, KRAS inhibition can induce adipogenic differentiation mediated by increased autophagy and thus enhance preadipocyte differentiation and lipid accumulation in mature adipocytes.

Then, we detected the effects of KRAS inhibition on the PI3K and MAPK pathways in 3T3-L1 and C2C12 cells. The PI3K pathway is an important pathway that mediates insulin functions. Its activation contributes to lipid accumulation [74,75]. The finding that the PI3K activator 740 Y-P increased TG levels is consistent with the important roles of PI3K signaling in lipid accumulation [76]. Surprisingly, our results showed that p-PI3K showed different expression patterns in 3T3-L1 and C2C12 cells, which may be due to the different cell types, and PI3K signaling was inhibited after KRAS inhibition. However, lipid accumulation was not negatively affected in either 3T3-L1 or C2C12 cells. On the one hand, this indicates that the activation level of PI3K does not play a major role in KRAS-mediated preadipocyte differentiation. On the other hand, it indicates that PI3K can still enhance differentiation in a manner unrelated to KRAS restriction. In addition, although the activity of PI3K was reduced by KRAS inhibition, PI3K reactivation helped to promote the differentiation of preadipocytes. This may be because adipogenic differentiation is regulated by complex molecular mechanisms, and KRAS also regulates other signal transduction pathways than the PI3K pathway. Therefore, we next determined the effects of KRAS on the activation levels of MAPKs.

The MAPK family, which includes three main important subfamilies (ERKs, JNKs, and p38s), is relatively conserved in mammals [22,77]. In human adipocytes and 3T3-L1 cells, MAPKs are involved in the regulation of lipid metabolism [78,79,80,81] and insulin resistance [82,83]. Our study showed that the phosphorylation level of ERK was significantly reduced after KRAS inhibition, indicating that KRAS may affect mitosis, the cell cycle, and cell proliferation in preadipocytes [84,85,86] by regulating the activation of ERK. In addition, the low activation level of ERK supports the notion that KRAS plays important roles in regulating the transcriptional activity of PPARγ, thereby regulating adipogenic differentiation [87,88]. This is also consistent with our finding that KRAS inhibition leads to cell growth arrest, promoting cell differentiation and lipid accumulation. Besides this, KRAS inhibition led to decreased p38 phosphorylation, which is consistent with the finding that increased phosphorylation of p38 inhibits adipogenesis [89,90]. The decreased phosphorylation level of JNK suggests that KRAS may inhibit adiponectin expression by regulating JNK and TNF-α, thereby inhibiting adipogenesis under physiological conditions [91] and affecting adipogenesis by regulating insulin resistance [92]. Moreover, KRAS inhibition can alleviate the inhibition of lipid accumulation induced by the MAPK activator ANI [93]. In addition, we tested the levels of RAS-GTP in 3T3-L1 and C2C12 cells after KRAS inhibition using a pull-down assay. RAS belongs to the GTPase family and thus is capable of hydrolyzing GTP. When RAS is bound to GDP, it is in an inactive state. When GEF converts GDP to GTP, RAS becomes active RAS-GTP, which regulates downstream pathway members such as RAF-MEK-ERK (MAPK), PI3K-AKT, and RAL-GEL. Our study showed that RAS-GTP levels decreased significantly after KRAS inhibition, which was consistent with our results showing that the phosphorylation levels of MAPK and PI3K in 3T3-L1 and C2C12 cells were decreased significantly after KRAS inhibition. Therefore, we propose that KRAS can inhibit adipogenic differentiation and lipid accumulation by maintaining or even inhibiting the phosphorylation levels of MAPKs.

According to previous studies, therapies and drugs used to treat disease should be developed from in vitro cell and animal experiments to clarify the specific mechanism, toxicity, and side effects before clinical tests. Overall, our data showed that KRAS, as an important regulator, affected pre-adipocyte differentiation and lipid accumulation, as well as cell proliferation and autophagy. These results will help us to explore the mechanism of preadipocyte differentiation, adipocyte maturation, and lipid accumulation. More importantly, KRAS may regulate MAPK and PI3K pathways. This makes KRAS a potential drug target to regulate obesity. In future work, we will try to synthesize molecular compounds which can target KRAS, and test whether they can be used to regulate the accumulation of fat in animal models. We hope our research could be used in actions against obesity.

**Figure 7:**
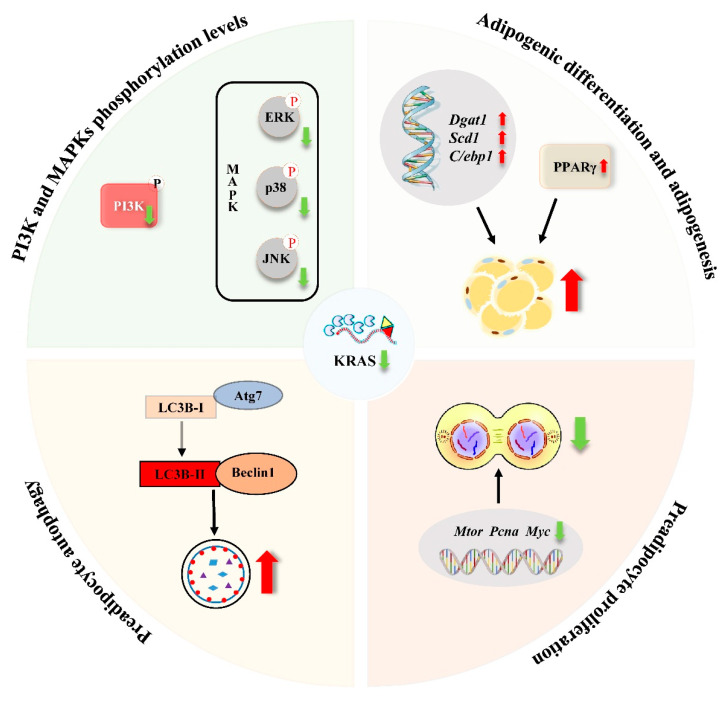
Schematic diagram of the effects of KRAS inhibition in 3T3-L1 and C2C12 cells. After KRAS inhibition, cell proliferation ability (*Mtor*, *Pcna*, and *Myc*) and phosphorylation levels of PI3K and MAPKs (ERK, JNK, and p38) were downregulated, while autophagy levels (LC3B puncta, *Atg7,* and *Beclin 1*) and adipogenic differentiation and adipogenesis (TG, *Dgat1*, *Scd1*, *C/ebp1,* and PPARγ) were up-regulated in 3T3-L1 and C2C12 cells.

In summary, KRAS affects cell proliferation, adipogenic differentiation, and lipid accumulation in 3T3-L1 and C2C12 cells by regulating autophagy, PI3K, and MAPK signaling (Figure 7). These findings help elucidate the potential role of KRAS in lipid metabolism and provide a theoretical basis for understanding the processes of lipid metabolism and the causes of obesity.

## 4. Materials and Methods

All reagents and chemicals were purchased from Sigma-Aldrich (St. Louis, MO, USA) unless indicated otherwise.

### 4.1. Cell Culture, Adipogenic Differentiation Induction, and Chemical Treatments

3T3-L1 mouse embryonic fibroblasts and C2C12 myoblasts were obtained from the National Collection of Authenticated Cell Cultures (Shanghai, China). The 3T3-L1 and C2C12 cells were cultured in high-glucose Dulbecco’s modified Eagle’s medium (DMEM, Gibco, Grand Island, NY, USA) supplemented with 10% fetal bovine serum (FBS, Biological Industries, Beit Haemek, Israel) at 37 °C in an atmosphere of 5% CO_2_. Adipogenic differentiation was initiated at day 0 by culturing the cells in high-glucose DMEM supplemented with 10% FBS, 5 μg/mL insulin (Solarbio, Beijing, China), 1 μM dexamethasone, and 0.5 mM 1-methyl-3-isobutylxanthine. A PI3K activator 740 Y-P (50 μg/mL, dissolved in dd H_2_O; MedChemExpress, NJ, USA) [76] or a MAPK activator anisomycin (ANI; 25 ng/mL, dissolved in DMSO; Selleck, Shanghai, China) [93,94] was added to the culture medium depending on the different experiment requirements. After 48 h (day 2), the cells were switched to DMEM supplemented with 10% FBS and 5 μg/mL insulin, and the medium was changed every 24 h for 6 days without 740 Y-P or ANI.

### 4.2. Cell Transfection

In brief, 4 μL each of 20 μM negative control (NC), siRNA (si-NC), or KRAS-targeting siRNA (si-KRAS) stock solution (GenePharma, Suzhou, China) was transfected with 2 μL Lipofectamine™ 2000 Transfection Reagent (Invitrogen, Rochester, NY, USA) into 3T3-L1 and C2C12 cells at a cell density of 70% in each well of 12-well plates (NEST Biotechnology, Wuxi, China). The final concentration of the siRNAs were 80 nM in high-glucose DMEM. After 6 h, the medium was changed to high-glucose DMEM with 10% FBS. Then, the culture plates were incubated in a 5% CO_2_ incubator at 37 °C for 18 h. The siRNA sequences are shown in Supplementary Table S1.

### 4.3. RAS-GTP Level Assay

The level of RAS-GTP was assessed using a RAS pull-down activation assay kit (NewEast Biosciences, Malvern, PA, USA, #81101), according to the manufacturer’s instructions. In brief, cells were cultured in 6-well plates to 70% confluence before transfection. Total cell lysates were collected and incubated with anti-active RAS monoclonal antibody plus protein A/G agarose bead slurry at 4 °C for 1 h with gentle agitation. Agarose beads were resuspended in 20 μL of 2× reducing SDS-PAGE sample buffer and boiled. Precipitated RAS-GTP was detected by Western blot analysis using anti-RAS monoclonal antibody (provided by the kit) according to the manufacturer’s instructions.

### 4.4. Autophagy Inhibition by Chloroquine

In brief, 3T3-L1 and C2C12 cells were cultured in 12-well plates until they reached 70% confluence. Then, cells were treated with 50 μm chloroquine (Selleck, dissolved in DMSO) for 24 h. Next, Western blot and immunofluorescence analysis were used to detect autophagy level.

### 4.5. RNA Extraction and Quantitative Real-Time Reverse Transcription Polymerase Chain Reaction (qRT-PCR)

3T3-L1 and C2C12 cells were washed once in 1× phosphate-buffered saline (PBS) and total RNA was extracted using TRIzol^®^ Reagent (Life Technologies, Carlsbad, CA, USA). First-strand cDNA was synthesized from 1 μg of total RNA using a fast reverse transcription kit (TIANGEN, Beijing, China) according to the manufacturer’s instructions. Each 20 μL qRT-PCR mixed system included 8 μL of deionized water, 10 μL of 2× SuperReal PreMix Plus (TIANGEN), 1 μL of cDNA, and 0.5 μL each of forward and reverse primers (10 μM). The qRT-PCR conditions included denaturation at 95 °C for 180 s and 40 cycles of 95 °C for 10 s, 60 °C for 20 s, and 72 °C for 30 s. Gene expression was quantified using the Mastercycler ep realplex (Eppendorf, Hamburg, Germany) and 2^−^^ΔΔCT^ method with GAPDH as the standard. The primer sequences are shown in Supplementary Table S2.

### 4.6. Protein Separation and Western Blot Analysis 

3T3-L1 and C2C12 cells were trypsinized, washed once in medium and once in PBS, and pelleted for 5 min at 500× *g*. Then, RIPA buffer (Solarbio) containing 1% PMSF (100 mM, Solarbio) was added to the cell suspension and mixed well. After that, the cells were ultrasonicated for 60 s (3 times per s). Next, the sample was lysed on ice for 30 min. After lysis, the mixture was centrifuged for 10 min at 16,000× *g* to collect the protein-containing supernatant. The samples and SDS-PAGE loading buffer (4×, with β-mercaptoethanol; Solarbio) were mixed and incubated at 95 °C for 5 min. The protein samples were separated by SDS-PAGE and then transferred to a polyvinylidene fluoride membrane (0.45 μm, Millipore, Bedford, MA, USA). Next, the membranes were transferred to 5% BSA blocking solution at room temperature, blocked for 2 h, and incubated with corresponding primary antibodies diluted to a suitable concentration with blocking solution at 4 °C overnight. After the membranes were washed 3 times with 1× Tris-buffered saline containing 0.5% Tween-20 (TBST) for 10 min each, the membranes were incubated at room temperature for 1 h with corresponding secondary antibodies. Finally, a Tanon 5200 image analyzer (Tanon, Shanghai, China) and ImageJ software (NIH, Bethesda, MD, USA) were used for image capture and gray value analysis. The GAPDH loading controls were performed on the same membrane. The antibodies used in the study are shown in Supplementary Table S3.

### 4.7. CCK-8 Assay

The detection of cell proliferation was carried out in accordance with the CCK-8 assay protocol (Beyotime, Shanghai, China). A cell suspension (2000 cells/well) was inoculated into clear-bottomed 96-well plates (Nest Biotechnology). After seeding and culturing, the cells (cell density was approximately 70%) were transfected with siRNA for 24 h. Then, 20 μL of CCK-8 solution was added to each well without changing the medium. The plates were incubated in a 5% CO_2_ incubator at 37 °C for 2 h, and the absorbance was measured at 450 nm using a microplate reader (BioTek Instruments, Winooski, VT, USA). 

### 4.8. 5-Ethynyl-2′-Deoxyuridine (EdU) Assay

Cell proliferation was assayed using the BeyoClick™ EdU-555 Cell Proliferation Detection Kit (Beyotime) according to the manufacturer’s instructions with some modifications. In brief, the cells were incubated in DMEM containing 10% FBS and 10 μM EdU (pre-balanced at 37 °C for more than 3 h) for 2 h. Next, the cells were fixed with 4% paraformaldehyde for 20 min and then permeabilized by incubation in PBS with 0.3% Triton X-100 for 20 min at room temperature. After washing three times with PBS, the cells were incubated with BeyoClick additive solution at room temperature in the dark for 30 min. Then, the cells were incubated with 10 μg/mL Hoechst 33342 at room temperature for 15 min to label the nuclei. An inverted research microscope (Ti2-U, Nikon, Tokyo, Japan) and ImageJ software (NIH, Bethesda, MD, USA) were used to analyze the number of EdU-positive cells and the total cell number. The proliferation rate was calculated as the ratio of EdU-positive cells to the total cell number. 

### 4.9. Triglyceride (TG) Assay

All experiments were performed according to the manufacturer’s instructions. The amount of intracellular TG relative to total protein was detected using a tissue/cell triacylglycerol assay kit (Applygen Technologies, Beijing, China) at 550 nm and a BCA protein assay kit (Beyotime) at 562 nm with a microplate reader (BioTek Instruments), respectively, according to the manufacturer’s instructions. The relative TG level was calculated as OD_TG-550_ values to total protein level.

### 4.10. (Oil red O) ORO Staining and Quantification

An ORO staining kit (Solarbio) was used to stain lipid droplets in mature 3T3-L1- and C2C12-derived adipocytes. In brief, cells were washed twice with PBS and fixed with ORO fixative at room temperature for 20 min. Then, cells were rinsed with 60% isopropyl alcohol and stained with ORO dye for 20 min. After the ORO dye was washed away with distilled water, ORO-stained cells were visualized with an inverted research microscope (Nikon). For quantification, intracellular ORO was extracted with 100% isopropanol and quantified by measuring the optical absorbance at 520 nm using a microplate reader (BioTek Instruments). Then, the total protein was detected by a BCA protein assay kit (Beyotime) at 562 nm with a microplate reader (BioTek Instruments). The relative ORO value was calculated as OD_ORO-520_ values to total protein level.

### 4.11. Immunofluorescence

Briefly, cell culture slides (Nest Biotechnology) were added to 6-well plates before cell seeding. Then, a total of 1 × 10^5^ cells were seeded on the slides with cell culture medium. After transfection, the cells were fixed in PBS containing 4% paraformaldehyde for 30 min and permeabilized with PBS containing 0.3% Triton X-100 at room temperature for 30 min. Then, cells were blocked in PBS containing 5% BSA at room temperature for 2 h. Next, the cells were incubated with related primary antibodies overnight at 4 °C. After washing three times with PBS, the cells were incubated at 37 °C for 1 h with related secondary antibodies. The cells were then stained with 10 µg/mL Hoechst 33342 for 10 min and washed three times with PBS. Finally, the cell culture slides were mounted onto the slides and examined under a confocal laser scanning microscope (Carl Zeiss, Jena, Germany). All antibodies used in the study are in shown in Supplementary Table S3.

### 4.12. Statistical Analysis

All experiments were performed three times unless indicated otherwise. Data are represented as means ± standard deviation. Data from two groups were compared using the Student’s *t*-test. Differences between three or more groups were analyzed using one-way analysis of variance (ANOVA). *p* values < 0.05 were considered as statistically significant. All statistical analyses were performed using SPSS software (version 21.0, IBM, Chicago, IL, USA). 

## Data Availability

Data is contained within the article or supplementary materials.

## Supplementary Materials

The following are available online at https://www.mdpi.com/article/10.3390/ijms222413630/s1.
