# Supplementary material for: KRAS Affects Adipogenic Differentiation by Regulating Autophagy and MAPK Activation in 3T3-L1 and C2C12 Cells
Source: Int J Mol Sci. 2021 Dec 20;22(24):13630. doi: 10.3390/ijms222413630 (PMC8707842; doi:10.3390/ijms222413630)
Supplement: Supplementary file 1 [file ijms-22-13630-s001.zip › ijms-1391825-supplementary.pdf]

International Journal of Molecular Sciences
MDPI

Supplementary Material

Supplementary Table S1. siRNA sequences used in this study

siRNA name	Sequence (5' to 3') [a]
siRNA-KRAS	Sense-GGACUCCUACAGGAAACAA***TT***
siRNA-KRAS	Antisense-UUGUUUCCUGUAGGAGUCC***TT***
siRNA-NC	Sense-UUCUCCGAACGUGUCACGU***TT***
siRNA-NC	Antisense-ACGUGACACGUUCGGAGAA***TT***

[a] Additional "***TT***" was added to 3'-end region to enhance the silencing effect and the stability of siRNA.

Supplementary Table S2. Primer sequences used in this study

Genes	Accession number	Primer Sequence (5' to 3') [a]	Product length (bp)
Gapdh	NM_001289726.1	F: GGAAGCTTGTCATCAACGGG<br>R: GGCGGAGATGATGACCCTTTT	176
Pcna	NM_011045.2	F: ATAAAGATGCCGTCGGGTGA<br>R: ATGTTCCCATTGCCAAGCTC	131
Mtor	NM_001386500.1	F: GCTACTGTGTCTTGGCATCC<br>R: CAGGAAAGGCATGACGAAGG	176
Myc	NM_001177352.1	F: AATCCTGTACCTCGTCCGATTCCA<br>R: TTTGCCTCTTCTCCACAGACACCA	195
Atg7	NM_001253717.2	F: GCCAACTCCACACTGCTTTC<br>R: TCTTCTGGGTCAGTTCGTGC	173
Beclin1	NM_001359819.1	F: GGCGGCTCCTATTCCATCAA<br>R: GGCAAGACCCCACTTGAGAT	96
Scd1	NM_009127.4	F: TGGGTTGGCTGCTTGTG<br>R: GCGTGGGCAGGATGAAG	150
Dgat1	NM_010046.3	F: TTCCGTCCAGGGTGGTAGT<br>R: GAATCTTGCAGACGATGGCAC	192
C/ebp-β	NM_001287738.1	F: GCAAGAGCCGCGACAAG<br>R: GGCTCGGGCAGCTGCTT	154
Hmgr	NM_001360165.1	F: GATTCTGGCAGTCAGTGGGAA<br>R: GTTGTAGCCGCCTATGCTCC	214

[a] F: Forward primer; R: Reverse primer.

International Journal of *Molecular Sciences*
MDPI

Supplementary Table S3. Antibodies used for Western Blot and immunofluorescence (IF)

**Primary antibodies**		
Antibody	Company[a]	Cat. Number
KRAS	ThermoFisher Scientific	415700
LC3B	Abcam	ab192890
p-PI3K	Cell Signaling Technology	17366S
PI3K	Abcam	ab74136
PPARγ	Cell Signaling Technology	2443S
ERK	Abcam	ab17942
p-ERK	Cell Signaling Technology	4370S
JNK	Abcam	ab179461
p-JNK	Abcam	ab124956
p38	Abcam	ab31828
p-p38	Cell Signaling Technology	4511S
GAPDH	Cell Signaling Technology	2118S
**Secondary antibodies**		
Antibody	Company[a]	Cat. Number
Goat anti-rabbit IgG	Bioworld Technology Inc.	BS13278
Goat anti-mouse IgG	Bioworld Technology Inc.	BS12478
Anti-rabbit IgG (for IF)[b]	Cell Signaling Technology	4412S

[a] ThermoFisher Scientific, Waltham, MA, USA.
Cell Signaling Technology, Beverly, MA, USA.
Abcam, Cambridge, MA, USA
Bioworld Technology Inc., Louis Park, MN, USA.
[b] Anti-rabbit IgG (H+L)-Alexa Fluor® 488 Conjugate.
